# Effect of Protective Layer on the Performance of Monocrystalline Silicon Cell for Indoor Light Harvesting

**DOI:** 10.3390/s23187995

**Published:** 2023-09-20

**Authors:** Tarek M. Hammam, Badriyah Alhalaili, M. S. Abd El-sadek, Amr Attia Abuelwafa

**Affiliations:** 1 Department of Physics, Faculty of Science, South Valley University, Qena 83523, Egypt; mahmoud.abdelsadek@sci.svu.edu.eg (M.S.A.E.-s.); amr.abuelwafa@sci.svu.edu.eg (A.A.A.); 2 Nanotechnology and Advanced Materials Program, Kuwait Institute for Scientific Research, P.O. Box 24885, Kuwait City 13109, Kuwait; 3 Department of Physics, Faculty of Science, Galala University, Suez 43511, Egypt

**Keywords:** encapsulation, lamination, monocrystalline, energy-harvesting solutions, indoor energy harvesting, smart buildings, protective layer

## Abstract

The development of renewable energy sources has grown increasingly as the world shifts toward lowering carbon emissions and supporting sustainability. Solar energy is one of the most promising renewable energy sources, and its harvesting potential has gone beyond typical solar panels to small, portable devices. Also, the trend toward smart buildings is becoming more prevalent at the same time as sensors and small devices are becoming more integrated, and the demand for dependable, sustainable energy sources will increase. Our work aims to tackle the issue of identifying the most suitable protective layer for small optical devices that can efficiently utilize indoor light sources. To conduct our research, we designed and tested a model that allowed us to compare the performance of many small panels made of monocrystalline cells laminated with three different materials: epoxy resin, an ethylene–tetrafluoroethylene copolymer (ETFE), and polyethylene terephthalate (PET), under varying light intensities from LED and CFL sources. The methods employed encompass contact angle measurements of the protective layers, providing insights into their wettability and hydrophobicity, which indicates protective layer performance against humidity. Reflection spectroscopy was used to evaluate the panels’ reflectance properties across different wavelengths, which affect the light amount arrived at the solar cell. Furthermore, we characterized the PV panels’ electrical behavior by measuring short-circuit current (I_SC_), open-circuit voltage (V_OC_), maximum power output (P_max_), fill factor (FF), and load resistance (R). Our findings offer valuable insights into each PV panel’s performance and the protective layer material’s effect. Panels with ETFE layers exhibited remarkable hydrophobicity with a mean contact angle of 77.7°, indicating resistance against humidity-related effects. Also, panels with ETFE layers consistently outperformed others as they had the highest open circuit voltage (V_OC_) ranging between 1.63–4.08 V, fill factor (FF) between 35.9–67.3%, and lowest load resistance (R) ranging between 11,268–772 KΩ.cm^−2^ under diverse light intensities from various light sources, as determined by our results. This makes ETFE panels a promising option for indoor energy harvesting, especially for powering sensors with low power requirements. This information could influence future research in developing energy harvesting solutions, thereby making a valuable contribution to the progress of sustainable energy technology.

## 1. Introduction

Energy security, a multifaceted concept affecting economics, the environment, and technology, has evolved into an interdisciplinary field due to climate change and fossil fuel uncertainties. Abundant and environmentally friendly renewable energy can mitigate energy shortages and support environmental conservation [1]. The increasing usage of energy-efficient electronic devices like sensors, remote controllers, electric decors, and Wi-Fi routers has increased demand for batteries. However, since these batteries have a limited lifespan, they can impact the longevity of wireless network nodes. Additionally, battery replacement incurs additional expense and waste.

On the other hand, indoor photovoltaics can provide an efficient and natural energy source for indoor applications. Due to their low cost and printable nature, indoor photovoltaic devices have the ability to fuel the Internet of Things (IoT) revolution [2]. Energy harvesting in indoor environments has become a promising field for developing efficient energy sources to build smart buildings. Photovoltaics, which are specifically tailored for low-density artificial light, have emerged as a prime candidate for this purpose. Therefore, the development of light-harvesting technology capability of producing extraordinary output power under low and indoor light conditions has enormous potential for use in domotics (home automation) and building management systems [3,4]. This technology could enable the development of self-powered and easy-to-install devices, such as intelligent sensors that can communicate wirelessly, which is considered the primary building block in the rapid expansion of the Internet of Things technology industry. Thus, reducing maintenance costs and increasing the flexibility of building management systems are essential, particularly without requiring modification of existing infrastructure. However, it can be difficult to accurately describe indoor illumination conditions, photovoltaic energy generation is considered a valuable option in outdoor and indoor settings.

Currently, the photovoltaic market for terrestrial applications is dominated by crystalline silicon, which offers a reasonable compromise between cost and performance. In 2021, module costs reached approximately 0.30 USD/W with efficiencies between 20% and 25% under direct solar irradiation [5]. In addition, the ability of a module to perform depends on how it reacts to changes in temperature, irradiance, spectral content, and nominal power that occur throughout the day and across the seasons [6]. 

During the past decade, several studies have analyzed the efficacy of various photovoltaic technologies under complete outdoor conditions. These photovoltaic cells are composed of materials such as copper indium gallium selenide (CIGS) [7], cadmium telluride (CdTe) [8], organic photovoltaics [9], crystalline silicon, amorphous silicon, dye-sensitized solar cell (DSC) and polymer photovoltaics [10,11]. Variables such as reflection, spectral response, irradiance, temperature, and nominal power output can affect the efficacy of these solar cell modules throughout the day and the year [12], researchers are trying to confront these environmental conditions, such as using nano-phase and phase change materials to reduce the temperature of photovoltaic panels [13]; therefore, it is important to analyze not just the overall power produced outside by different panels over time, but also how performance changes and responds to different factors under diverse operating environments, including inside [14,15]. 

Although crystalline silicon cells continue to dominate and lead the market for internal and external solar cells depending on many scientific factors [16], including reliability and longevity [17], relatively higher efficiencies that typically range from 15% to 25% [18], availability due to being one of the most abundant elements on Earth [19], scalability, and versatility since they are integrated into a variety of external and internal applications, such as roofing systems and small devices [20], Since encapsulation is a crucial stage in the production of solar panels and an influencing factor on their performance, this effect must be studied, requiring to study a variety of materials that use as a protective layer. Polyethylene terephthalate (PET) is a common material known for its exceptional transparency and durability. PET provides a barrier against environmental elements such as moisture and ultraviolet rays while permitting sunlight to reach the solar cells [21]. Ethylene tetrafluoroethylene (ETFE) is another material used in solar panel lamination; it is extremely transparent, lightweight, and resistant to severe weather conditions. ETFE is known for its long-term stability and can improve solar panel performance and durability [22]. Epoxy, a thermosetting polymer, is also used as an encapsulation material in some solar panels due to its superior adhesion properties and high mechanical strength. Epoxy encapsulation can provide protection against moisture, chemicals, and ultraviolet light, making it suitable for use in challenging environments [23,24]. Overall, the lamination of PV panels with PET, ETFE, or epoxy as the first layer protects them and ensures their long-term performance and durability.

Researchers tried to study the effect of these protective layers on the PV panel performances, Acevedo-Gómez et al., tested the energy loss due to encapsulating monocrystalline cells with epoxy and compared it to others not coated with any material [25]. Lisco, F. et al., tested some optical and chemical properties of ETFE as a protective layer for the solar cell. Still, the electrical behavior and parameters such as V_OC_ or FF were not studied [26]. Kirpichnikova et al., present an investigation into using an innovative heat-resistant film that utilizes holographic coating and a total internal reflection prism layer [27]. Castro-Hermosa et al., tested perovskite cells coated with a layer of PET/ITO and ultra-thin flexible glass (FG) for indoor harvesting [28]. Some studies compared different cell technologies without considering the packaging material of these cells and the extent of their effect. Therefore, we found a gap in studying cells available on the market and ready for actual use and integration into internal power supply systems and the effect of the protective layers on electrical behavior and their parameters.

This paper offers a realistic analysis of internal light harvesting through small crystalline silicon PV panels. The focus was on monocrystalline cells for their higher efficiency than polycrystalline cells [29], as both are available in the market and at prices. To measure photovoltaic performance at various levels of illumination, a photovoltaic testing station was set up indoors, so it could be outfitted with various artificial illumination sources. The two most popular lighting options today, compact fluorescent lights (CFL) and light-emitting diodes (LED), were chosen. The PV panels’ performance was evaluated at various illumination levels using CFL and LED. Even though the small PV panels were intended for interior uses, they may still be exposed to various environmental factors over time, including humidity, changes in temperature, and dust buildup, which can reduce the PV panels’ efficiency. Measuring the contact angle can provide information about the wettability of the surface and the hydrophobic or hydrophilic nature of the protective layer, which can influence the PV panels’ resistance to moisture, dust, and other environmental factors and help determine its long-term durability.

## 2. Materials and Methods

### 2.1. PV Panels

**Figure 1:**
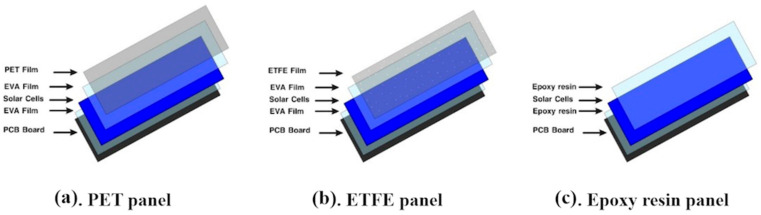
The structure of each monocrystalline PV panel. (**a**) a PET panel that consists of a solar cell between two layers of EVE above the PCB board and laminated with a PET film; (**b**) an ETFE panel that consists of a solar cell between two layers of EVE above the PCB board, and laminated with a film of ETFE, (**c**) an Epoxy resin panel that consists of two layers of epoxy encapsulating the solar cell above the PCB board.

Monocrystalline silicon solar cells’ stability, affordability, and overall efficacy make them an excellent option for indoor energy harvesting applications. In this research, Monocrystalline panels were customized in Ningbo Yangjiang Senzhou Photovoltaic Co.—Ningbo, China with the same dimensions of 5 × 5 cm^2^, thicknesses of 2.5 mm, a weight of 8.0 g, and an active area of 1.59 cm^2^, with 0.5 mm different Protective layers such as epoxy resin, ethylene–tetrafluoroethylene copolymer (ETFE), and polyethylene terephthalate (PET) Five panels from every type. From a structural view, the common elements across the three structures include the existence of a PCB board, while in ETFE and PET panels, the solar cells are encapsulated with an EVA, which is considered the most used encapsulant material in the PV industry. ETFE and PET sheets were extruded to laminate the cells and work as protective layers without any air gaps between the layers, as shown in Figure 1a,b, while in the epoxy panel, the solar cell was sandwiched by epoxy resin and also used as a Protective layer at the same time, as shown in Figure 1c. 

### 2.2. Contact Angle (OCA)

Using the optical contact angle (OCA) instrument from DataPhysics Instruments GmbH Co.—Filderstadt, Germany, the contact angles of the three different protective layers, ETFE, epoxy resin and PET, were measured on solar panels. Before the measurements of the contact angles, the solar panels were meticulously cleaned with ethyl alcohol to remove any surface contaminants. Then, to measure the contact angles, droplets of deionized water (2 μL) were meticulously dispensed onto the surface of each protective layer using a micropipette, and the OCA device’s high-resolution camera was used to capture the shape and size of the droplets. The obtained images were then analyzed with specialized image analysis algorithms in order to calculate the contact angles.

### 2.3. Reflection Spectroscopy

UV-670 UV-VIS Spectrophotometer from Jasco was used to measure the reflection intensity for the three solar panels with different protective layers. An integrating sphere accessory was connected to the UV-670 in order to utilize it to measure reflectance. The integrating sphere holding the solar panels would gather the reflected light and send it to the spectrophotometer. The computation of reflectance would therefore be possible owing to the spectrophotometer’s measurement of the intensity of the reflected light at each wavelength.

The UV-670 UV-VIS 190–2700 nm wavelength range is more than adequate to measure reflectance at the experiment’s target wavelengths ranging from 200–1000 nm. Furthermore, the UV-670 UV-VIS spectral bandwidth of 1.8 nm enables accurate reflectance measurement with a 5-nanometer step size. The instrument is a good choice for this experiment because it also has an easy-to-use software package that analyzes the reflectance data obtained for each solar panel.

### 2.4. Characterization Measurement System

**Figure 2:**
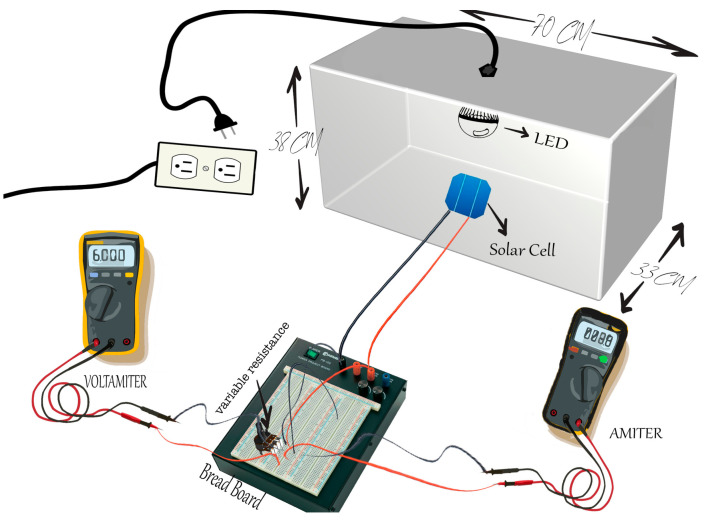
A 3D schematic illustrates the connections of the photovoltaic (PV) panel, positioned under the light source within the enclosed black box, to a variable resistance, ammeter, and voltameter, arranged on a breadboard to make up an indoor electrical characterization measurement system.

**Figure 3:**
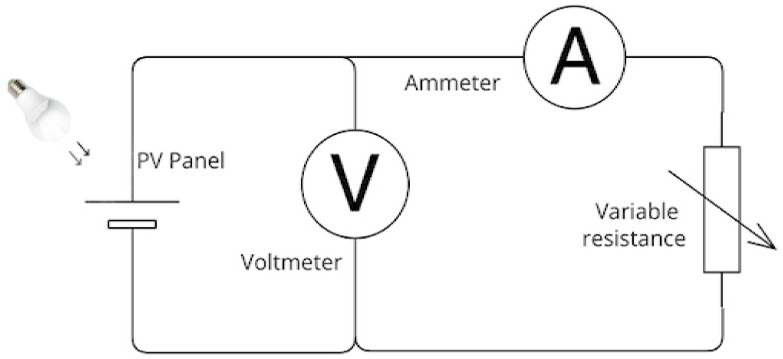
A circuit diagram used in the electrical characterization measurement system consists of a PV panel, a voltmeter, an ammeter, and a variable resistance.

The test model was designed and implemented in a custom black box for artificial interior lighting. An E14 bulb holder was inserted at the top of the box, as shown in Figure 2. Since the most frequently used light sources for indoor spaces are LED bulbs and cathode fluorescent lamp (CFL) bulbs [30], I-V measurements of common light sources in the local market are CFL (CHAOYI 85W and OSRAM 23W) and LED (MEXTOH 3W, 9W, 12W, and AKT 24W). The illuminance is determined by a simple measurement illuminometer (TASI, model TA8121 with 0.1 Lux resolution, overall accuracy ±3% rdg ±10 dgts) designed by Suzhou TASI Electronics Co. (Suzhou, China), that is because the irradiation from an artificial light source is usually measured in photometric measures (Lux, illuminance) rather than radiometric values (W/m^2^, irradiance), which represents the intensity of light as seen by the human eye [31]. The measurement process is carried out by placing the PV panel in its place inside the black box and connecting it to the electrical circuit, which is diagramed in Figure 3. The measurement system consists of a breadboard with variable resistance and is connected to a voltmeter and an ammeter, while the load voltage changes by raising the resistance values from 0–500 KΩ, and the voltage and current readings are recorded in a datasheet, using originPro software 2022 we drawing this data as the I-V curves for each PV panel under different light sources.

## 3. Results and Discussion

### 3.1. Contact Angle Comparison: OCA Results

**Figure 4:**
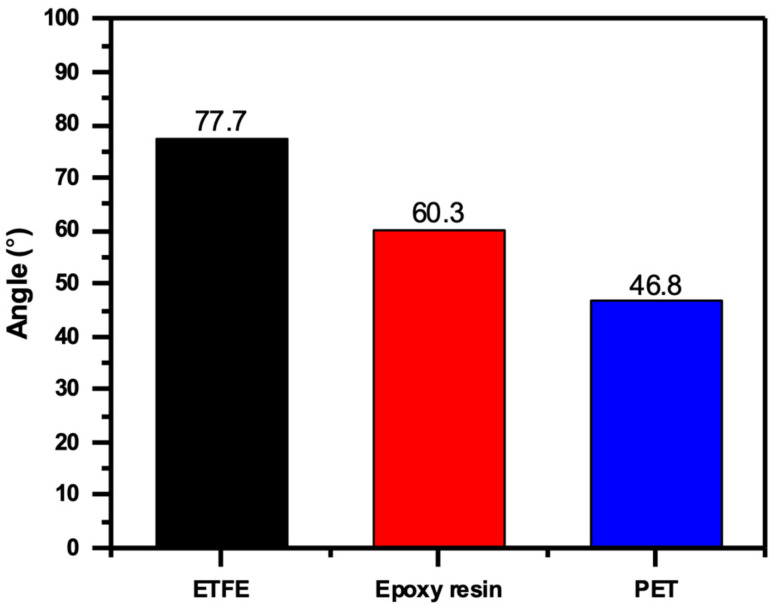
A histogram of the differences in the mean values of the measured contact angles (CA) for the ETFE, epoxy resin, and PET panels.

**Figure 5:**
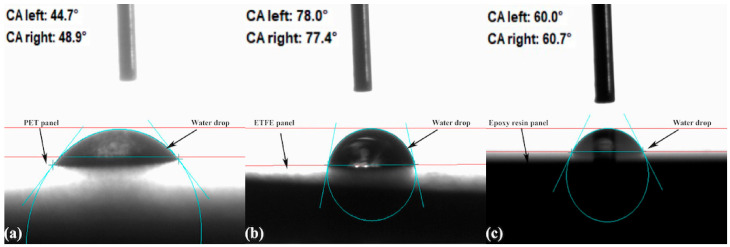
Water droplets on the surfaces of (**a**) PET panel, (**b**) ETFE panel and (**c**) Epoxy resin panel as captured from OCA camera.

Using the OCA device, measurements of the contact angles of the three PV panels with various protective layers (ETFE, epoxy resin, and PET) revealed significant differences. Figure 4 displays a three-column histogram representing each type’s mean degrees of the right and left contact angles (an average of five samples of each type was taken). In addition, images of the water droplets on the surfaces of the three panels, obtained via OCA measurements at home temperature, are depicted in Figure 5.

ETFE layer showed the highest mean contact angle of 77.7°, indicating a highly hydrophobic surface. This is likely due to the unique properties of ETFE, which is known for its excellent water repellency, low surface energy, and potential for self-cleaning properties [32]. The average contact angle for the epoxy resin layer was 60.3°, indicating a relatively hydrophobic surface. In contrast, the PET layer exhibited the lowest contact angle with a mean value of 46.8°, indicating a less hydrophobic surface than the other two materials. 

The significant difference in the contact angles between the three protective layers indicates that the surface properties of the materials play an essential role in determining their wettability and hydrophobicity. The higher contact angle value of the ETFE layer may indicate enhanced water repellency, which could prevent water ingress and degradation of the PV panel. With a moderate contact angle, epoxy resin may offer a hydrophobicity that is suitable for certain applications. The PET layer, which has the smallest contact angle, may have a comparatively lower hydrophobicity, which could affect its performance in environments prone to moisture.

Our findings provide valuable insights into the suitability of these materials for various applications. Table 1 below presents a comparison of our results with those from other studies.

The table compares contact angles for various protective layers used on various PV panel technologies. Glass/ITO displays a somewhat hydrophilic surface with a contact angle of 29° in the context of perovskite solar cells (PSC), while PET/ITO demonstrates a highly hydrophobic surface with a contact angle of 93° and FG/ITO displays a moderately hydrophobic surface with a contact angle of 60°. On the other hand, the surface of polycrystalline silicon panels made of ETFE has a contact angle of 110° and is extremely hydrophobic. Our study reveals that epoxy resin demonstrates a moderately hydrophobic surface at 60.3°, whereas PET exhibits a lower contact angle of 46.8°, indicating reduced hydrophobicity. However, the ETFE layer, when applied to monocrystalline silicon PV panels, exhibits a contact angle of 77.7°. This finding aligns with the hydrophobicity reported in previous research [33], suggesting that ETFE retains its excellent self-cleaning properties when used as a protective layer on this type of PV panel technology. These different contact angles show the varying levels of hydrophobicity among protective layers. This offers important information for selecting materials based on specific PV panel technologies and intended applications, especially regarding moisture resistance and long-term performance.

### 3.2. Reflection Mesurments

**Figure 6:**
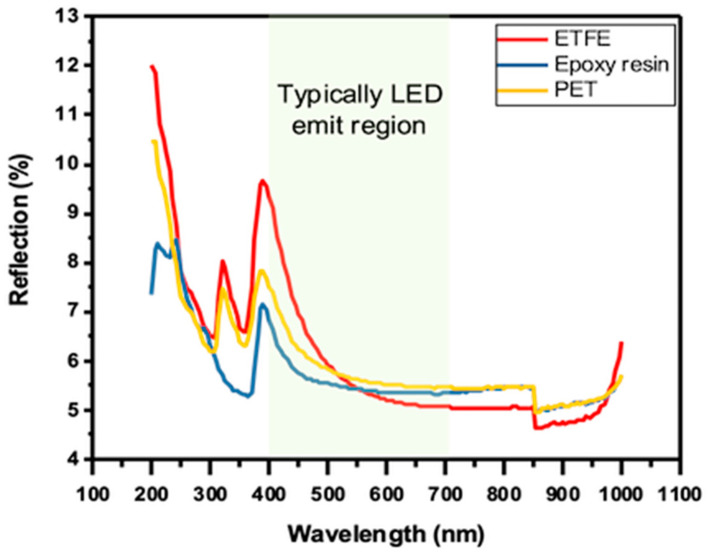
The reflection spectrum of PET, ETFE, and Epoxy resin protective layer PV panels in the wavelength range of 200–1000 nm, and the shaded area shows the behavior of each PV panel reflection in the typically LED emit region between 400 and 700 nm.

Reflection (%) versus wavelength (nm) plots show the results of spectroscopic reflection measurements of three solar panels with various protective layers, as shown in Figure 6. It was observed that the reflectance properties of the solar panels vary substantially depending on their protective layer.

The relevant spectral response range between 350 and 1100 nm for common c-Si solar cell energy conversion covers 99% of the AM 1.5 spectrum [34]. The solar panel encapsulated with epoxy resin exhibited the lowest reflectance values, ranging from 4.95% at 850 nm to 7.15% at 390 nm and averaging at 5.47%. On the other hand, the solar panel laminated with ETFE showed intermediate reflectance values, ranging from 4.63% at 860 nm to 9.66% at 390 nm, with an average reflection intensity of 5.68%. The solar panel laminated with PET showed the highest reflectance values, ranging from 4.95% at 855 nm to 7.8% at 385 nm and an average of 5.7%.

The results indicate that the choice of protective layer material can substantially impact the reflectance properties of solar panels. The PV panel with a PET layer exhibited the highest reflectance, which could result in greater energy loss due to increased light reflectance. PV panel with an epoxy resin layer, on the other hand, exhibited a reduced reflectance, which could result in greater light transmission and potentially greater solar panel energy conversion efficiency. PV panel that has an ETFE layer, exhibited moderate reflectance properties. These results emphasize the significance of protective layer material selection for solar panel performance, and further research is required to optimize protective layer materials for enhanced solar panel performance. However, if we consider the typical LED bulb, it emits light with wavelengths between 400–700 nanometers, which correspond to the visible light spectrum [35,36]. PV panel with an epoxy resin layer exhibited the lowest percentage of reflection across the entire wavelength range from 400 nm to 700 nm, with values ranging from 5% to 9.3%. With an average value of 5.56%, The PV panel with a PET layer showed a slightly higher reflection percentage than the PV panel with an epoxy resin layer, ranging from 5.4% to 7.5%, with an average of 5.85%. PV panel that has an ETFE layer had the highest percentage of reflection, ranging from 5% to 9.3% and averaging at 5.95%.

### 3.3. Electrical Charectraization 

#### 3.3.1. Current–Voltage Analysis under Light Illumination of Different Intensities

**Figure 7:**
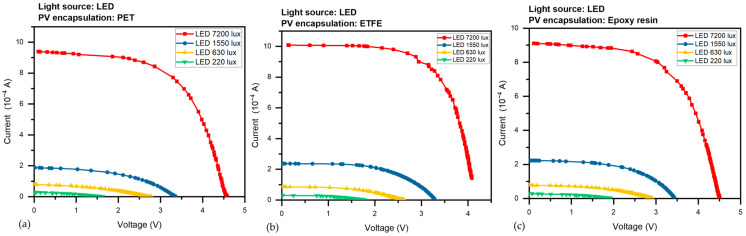
The I-V curves for the three PV panels under LED illumination were (**a**) I-V for the PET panel, (**b**) curves for the ETFE panel and (**c**) for the epoxy panel.

**Figure 8:**
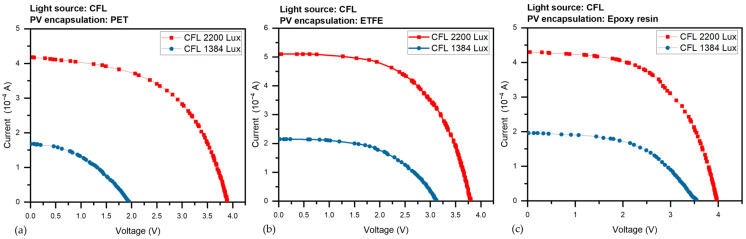
The I-V curves for the three PV panels under CFL illumination were (**a**) I-V for the PET panel, (**b**) curves for the ETFE panel and (**c**) for the epoxy panel.

The devices were subjected to various light illuminations of the LED from 220 to 7200 Lux, and with two CFL illumination intensities (1384 and 2200 Lux), we gathered a set of current-voltage (I-V) curves for the three PV devices. the amount of photocurrent produced at 0 V causes the I-V curve to shift downward, signifying I_sc_. The short-circuit current (I_sc_) is the maximum current without voltage, while the open-circuit voltage (V_oc_) is the maximum voltage without current. Both V_oc_ and I_sc_ vary with light intensity. Figure 7 presented the average corresponding results under LED light were (a) I-V for the PET panel, (b) curves for the ETFE panel and (c) for the epoxy panel, as the panels take the same behavior under CFL light in Figure 8a–c.

The output power is also calculated from the I-V datasheet according to Equation (1).

(1) P=I×V

where P is power, I refer to Current, and V is voltage.

This enables us to obtain the P-V curves for each panel in every illumination level, which is important to determine P_max_, the highest point of the P-V curve.

**Figure 9:**
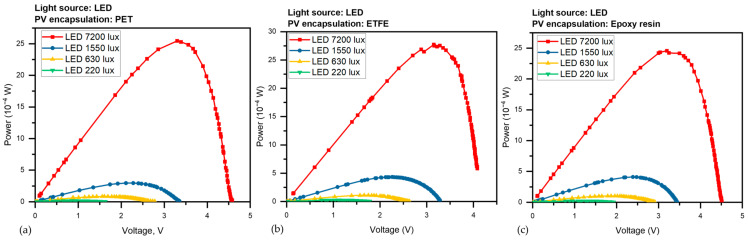
Output power P versus voltage V of the three PV panels under LED, (**a**) P-V for the PET panel, (**b**) curves for the ETFE panel and (**c**) for the epoxy panel.

**Figure 10:**
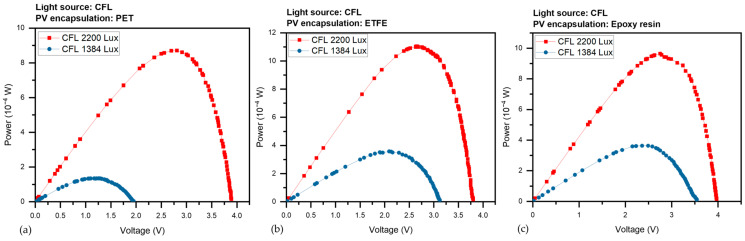
Output power P versus voltage V of the three PV panels under CFL, (**a**) P-V for the PET panel, (**b**) curves for the ETFE panel and (**c**) for the epoxy panel.

Regardless of the type of light source, Figure 9a–c depicts the output power P versus voltage V of the three different PV panels under various LED illumination intensities (220–7200 Lux). Figure 10a–c, depict the output power, P, versus voltage, V, of the three different PV panels under CFL at two various illumination intensities (1384 and 2200 Lux). Maximum electric power (P_max_) is the utmost power at the operating point of the panel, which equals (I_max_ × V_max_). It is evident from Figure 7 that all power constraints are increased by light intensity.

According to Figure 9 and Figure 10, the P-V feature is used to specify the maximum voltage (V_max_) and the maximum power (P_max_) that the photovoltaic panels can generate for each illumination level. The photocurrent phenomenon, which is an increase in current with illumination, is thought to be a sign of the free carriers produced by the absorbed photons. The absorption of photons and the photogeneration of charge carriers are two of the mechanisms involved in the photocurrent phenomenon [37,38].

The maximum photocurrent and maximum photovoltage may be calculated from the P-V curve by using the previously mentioned I-V characteristic.

(2) $$P_{\max}=I_{\max}\times V_{\max}$$

where P_max_ is the maximum power output, I_max_ is the current at the maximum power output, and V_max_ is the voltage at the maximum power output.

These variables allow for the following calculation of the photovoltaic/photosensor cell fill factor (FF) [38,39].

(3) $$\mathrm{FF}=P_{\max}/(I_{\mathrm{sc}}\times V_{\mathrm{OC}})$$

FF, the fill factor, describes the ratio of maximum power obtainable of the PV module’s short-circuit current I_sc_ and open-circuit voltage V_oc._

Ohm’s law was used to determine the load resistance corresponding to the P_max_ [39]:

R = V_max_/J_max_ (4)

J_max_ = I_max_/A (5)

where A represents the area in cm^2^, and J_max_ refers to the current density.

#### 3.3.2. Comparison of PV Panels

There is no doubt that the power output of a PV panel is influenced by several factors, including the spectral composition of the incident light, its intensity [40,41], and its angle [42]. As a result, the performance of a photovoltaic device needs to be optimized according to its ultimate application and vice versa; the appropriate application for a photovoltaic device with a certain spectral response should also be selected. In light of this, the main performance parameters, including short-circuit current (I_sc_), open-circuit voltage (V_oc_), maximum power output (P_max_), load resistance (R), and fill factor (FF), were measured under the various intensities of two light sources, LED and CFL. The histograms showing the differences in I_sc_, V_oc_, P_max_, R, and FF for each form of the protective layer under each illumination will be compared and discussed.

In the beginning PV panel with PET layer parameters is presented in Table 2, in order of importance, V_oc_ (open-circuit voltage), I_sc_ (short-circuit current), P_max_ (maximum power), FF (fill factor), and load resistance (R) values for the panel with PET layer were measured under various lighting conditions. I_sc_ increased from 0.28 × 10^−4^ A to 9.39 × 10^−4^ A, V_oc_ increased from 1.63 V to 4.58 V, P_max_ increased from 0.16 × 10^−4^ W to 25.4 × 10^−4^ W, and R decreased from 14,398 to 944 KΩ.cm^−2^ when the light intensity increased from 220 Lux to 7200 Lux. However, as illumination increased, the FF further demonstrated an upward trend from 35.9% to 59%. However, The PV panel with an ETFE layer exhibits a marginally higher FF than panels with PET and epoxy resin layers, especially at higher illumination levels.

Similar trends were seen in Table 3 with The PV panel with the ETFE layer, which increased I_sc_ from 0.3 × 10^−4^ A to 10.09 × 10^−4^ A, V_oc_ from 1.63 V to 4.08 V, and P_max_ from 0.25 × 10^−4^ W to 27.7 × 10^−4^ W, and R (load resistance) decreased from 11,268 to 772 KΩ.cm^−2^ with an increase in illumination from 220 Lux to 7200 Lux. Along with an increase in lighting, the FF also increased, going from 35.9 to 67.3%. The PV panel with the ETFE layer performs consistently better than the other two protective layers, with higher I_sc_ values at all illumination levels. 

As illumination increased from 220 Lux to 7200 Lux, I_sc_ for the PV panel with the epoxy resin layer climbed from 0.28 × 10^−4^ A to 9.11 × 10^−4^ A, V_oc_ grew from 1.93 V to 4.51 V, and P_max_ increased from 0.23 × 10^−4^ W to 24.58 × 10^−4^ W; R (load resistance) decreased from 17,048 to 934 KΩ.cm^−2^. FF slightly increased, from 42.3 to 59.8%, as shown in Table 4. The PV panel with the epoxy resin layer has marginally higher V_oc_ values than the PV panel with PET and ETFE layers. However, the variance in V_oc_ values is not statistically significant at all illumination levels.

At the same point, the performance of the ETFE laminated panel was better than the other two panels, especially under high illuminations in the two types of lighting used in the experiment, as it produced high external power, as seen in the P_max_ values; on the other hand, panels laminated with PET and epoxy resin were close at all low-illumination levels, with a slight superiority for the epoxy resin panels, but this behavior changed in favor of panels with PET until they outperformed the epoxy resin cells at high illumination levels.

Another point of comparison between our PV panels was the load resistance, the values of which establish a PV panel’s maximum power output for a specific lighting intensity, which substantially impacts the panel’s overall performance. Higher load-resistance values correspond to a panel of higher voltage, whereas lower load-resistance values correspond to a panel of higher current. Between the three distinct protective layer options and with changes in illumination intensity, the load-resistance values vary considerably. For instance, panels laminated with epoxy resin need a load resistance of 17,048 KΩ.cm^−2^ to achieve maximum power production under low illumination levels of 220 Lux, whereas panels laminated with PET and ETFE layers only need significantly lower values of 14,398 and 11,268 KΩ.cm^−2^, respectively. Under higher illumination levels of 7200 Lux, the load-resistance values for all varieties of protective layers decrease substantially, with PET, ETFE, and epoxy resin layers requiring values of 944, 772, and 934 KΩ.cm^−2^, respectively. 

For further clarification, Table 5 displays the range of performance parameters for each PV panel protective layer material. These ranges emphasize the variability of performance parameters among the various protective layer materials and provide valuable information for selecting the most appropriate PV panel for particular applications and lighting conditions.

Finally, the performance of the cells presented in this work was very good when compared to the silicon cells manufactured in reference [27], especially if one considers the difference in technology, and the glass packaging that increases weight and hinders use, which demonstrates their potential for integration into internal systems and providing energy for electronics and sensors. Also, Table 6 presents data on current efforts to use and test other technologies in this field. Although some studies have reported the good performance of these technologies, all of these studies are still within the scope of the laboratory due to issues of toxicity, stability, and performance in the long term. There is a need for research and development in this field in many ways to improve the performance of technologies and explore other protection layers.

## 4. Conclusions

This study explored the electrical properties of small optical devices appropriate for energy harvesting indoors. Monocrystalline cells panels laminated with three distinct substances, namely epoxy resin, an ethylene–tetrafluoroethylene copolymer (ETFE), and polyethylene terephthalate (PET), were examined under various light sources, including light-emitting diodes (LED) and compact fluorescent lamps (CFL). Panels laminated with an ETFE layer demonstrated the maximum open circuit voltage (V_oc_) and fill factor (FF) across a range of light intensities, making them a promising option for indoor energy harvesting. The study’s findings shed light on the optimal protective layer and light source for small optical devices for sustainable and reliable indoor energy harvesting. The panels’ contact angles and reflectance properties for each protective layer material were also discussed. Panels laminated with the ETFE layer exhibited the greatest contact angle, indicating enhanced water repellency, whereas panels laminated with the PET layer exhibited the least hydrophobicity. Panels laminated with the epoxy resin layer exhibited the lowest reflectance, allowing for greater light transmission and possibly a higher solar panel energy-conversion efficiency. However, the panel with the PET protective layer had the maximum reflectance, resulting in greater energy loss as a result of increased light reflectance. Overall, the selection of the protective layer material significantly impacts the wettability, reflectance, and efficacy of the panel. The study’s results provide pertinent photovoltaic parameters, including the short-circuit current, open-circuit voltage, maximal power output, load resistance, and fill factor for each panel under different lighting conditions. All panels experience an increase in I_sc_ (short-circuit current), V_oc_ (open-circuit voltage), and P_max_ (maximum power) as illumination increases. In contrast, load resistance values diminish, indicating a higher current discharge or higher voltage output. The panel laminated with epoxy resin layer has slightly higher V_oc_ values than the panels laminated with PET and ETFE layers, but the panel laminated with ETFE layer performs better under intense illumination. The P_max_ values for panels with PET and epoxy resin layers may be close at all illumination levels. The load resistance values for the three-panel options vary significantly, with epoxy resin requiring the highest values and with ETFE requiring the lowest. Overall, the article provides insightful information regarding the performance of various protective layer options for PV panels under variable illumination levels. Based on the desired current discharge or voltage output, the findings could be utilized to select the most suitable protective layer material for a particular PV panel application.

## 5. Recommendations and Future Work

ETFE layer preference: These panels demonstrated superior open-circuit voltage (V_oc_) and fill factor (FF) across various light intensities, enhancing performance. ETFE’s high contact angle also suggests improved resistance to moisture-related issues; hence, we expect it to be suitable for long-term use. So, the study recommends panels laminated with ethylene–tetrafluoroethylene copolymer (ETFE) for indoor energy harvesting applications.

Real-life testing: The process of selecting layers for panels involves making choices, as each material has its own expected challenges when used in real-life applications. Epoxy resin, although cost-effective and easy to apply, can face issues, like degradation from UV rays and becoming brittle over time, which can potentially affect its long-term durability. On the other hand, ETFE offers durability and allows light transmission but might require specialized installation and maintenance to address concerns such as sensitivity to scratches and the buildup of electrostatic charges. PET, renowned for its transparency and flexibility, is susceptible to problems like UV degradation, absorption of moisture and mechanical damage. These challenges emphasize the importance of research and development efforts aimed at improving the performance and sustainability of layers for solar panels while effectively adapting these materials to real-world conditions. So, we plan to integrate these devices into small-scale indoor applications such as sensors or low-power devices within our institute and study them for a long time.

Exploring and modification: Exploring alternative protective layer materials or modifications to the available materials may enhance the efficiency and cost of the panels.

Environmental impact assessment: Future research will focus on assessing the effects of many environmental variables, including temperature fluctuations, dust accumulation, and shadowing, on the efficacy of protective coatings in indoor energy harvesting panels. This encompasses examining the impact of temperature variations on the resilience and adhesion of protective layers, as well as the investigation into how dust or partial shadowing affects their capacity to transmit light. Comprehending these environmental impacts is vital in order to develop protective layers that can endure indoor settings and sustain their efficacy over an extended duration.

Comparative analysis: Comparative analyses will be conducted with other indoor energy-harvesting technologies, including different PV panels. These comparisons will provide valuable insights and benchmarking data, contributing to advancements in the field and helping identify the most effective solutions.

In summary, the research suggests that ETFE-laminated panels are a favorable option for indoor energy harvesting owing to their superior performance. To contribute to advancing the field of indoor energy harvesting, future research will involve real-life testing, the optimization of protective layers, efficiency enhancements, an environmental impact assessment and comparative analyses.

## Figures and Tables

**Table 1:** Comparison of Contact Angles for Various Protective Layers on PV Panels.

Layer	Technology	Contact Angle (°)	Reference
Glass/ITO	PSC	29	[27]
PET/ITO	PSC	93	[27]
FG/ITO	PSC	60	[27]
ETFE	Poly c.Si	110	[33]
Epoxy	Mono c.Si	60.3	This work
PET	Mono c.Si	46.8	This work
ETFE	Mono c.Si	77.7	This work

**Table 2:** Shown parameters of panel with PET protective layer.

Light Source	Illumination, Lux	I_sc_ (10^−4^, A) ± 0.04	V_oc_, V ± 0.09	P_max_ (10^−4^ W)	FF, %	I_max_ (10^−4^, A)	V_max_, V	J_max_, (A/cm^2^)	R (KΩ.cm^−2^)
LED	7200	9.39	4.58	25.4	59.2	7.71	3.3	0.00485	944
LED	1550	1.88	3.35	2.96	47	1.49	2.26	0.00094	3575
LED	630	0.78	2.78	0.86	39	0.54	1.56	0.00034	8185
LED	220	0.28	1.63	0.16	35.9	0.18	0.91	0.00011	14,398
CFL	2200	4.18	3.89	8.71	53.6	3.1	2.81	0.00195	1995
CFL	1384	1.68	1.95	1.35	41.2	1.07	1.26	0.00067	2898

**Table 3:** Shown parameters of panel with ETFE protective layer.

Light Source	Illumination, Lux	I_sc_ (10^−4^, A) ± 0.06	V_oc_, V ± 0.15	P_max_ (10^−4^ W)	FF, %	I_max_ (10^−4^, A)	V_max_, V	J_max_, (A/cm^2^)	R (KΩ.cm^−2^)
LED	7200	10.09	4.08	27.7	67.3	8.4	3.28	0.00528	772
LED	1550	2.37	3.28	4.3	55.8	1.92	2.26	0.00120	2716
LED	630	0.85	2.63	1.1	49.8	0.67	1.66	0.00042	6241
LED	220	0.3	1.63	0.25	35.9	0.23	1.1	0.00014	11,268
CFL	2200	5.1	3.8	11.1	57	4.17	2.65	0.00262	1449
CFL	1384	2.15	3.12	3.6	53.3	2.15	2.09	0.00135	2307

**Table 4:** Shown parameters of panel with epoxy resin protective layer.

Light Source	Illumination, Lux	I_sc_ (10^−4^, A) ± 0.03	V_oc_, V ± 0.1	P_max_ (10^−4^ W)	FF, %	I_max_ (10^−4^, A)	V_max_, V	J_max_, (A/cm^2^)	R (KΩ.cm^−2^)
LED	7200	9.11	4.51	24.58	59.8	7.68	3.2	0.00483	934
LED	1550	2.23	3.44	4.11	53.6	1.72	2.39	0.00108	3180
LED	630	0.78	2.91	1.04	45.7	0.58	1.79	0.00036	7977
LED	220	0.28	1.93	0.23	42.3	0.18	1.27	0.00011	17,048
CFL	2200	4.3	3.97	9.65	56.5	3.51	2.75	0.00221	1798
CFL	1384	1.96	3.54	3.64	52.4	1.46	2.49	0.00092	3855

**Table 5:** The range of parameters for each PV panel.

Protective Layer	I_sc_ (×10^−4^ A)	V_oc_ (V)	P_max_ (×10^−4^ W)	FF (%)	R (KΩ.cm^−2^)
PET	0.28–9.39	1.63–4.58	0.16–25.4	35.9–59	14,398–944
ETFE	0.3–10.09	1.63–4.08	0.25–27.7	35.9–67.3	11,268–772
Epoxy resin	0.28–9.11	1.93–4.51	0.23–24.58	42.3–59.8	17,048–934

**Table 6:** Current efforts in indoor energy harvesting technologies.

Front Layer	Technology	Ev (lx)	Voc (V)	FF, %	Area (cm^2^)	Fabrication	Reference
PET	PSC	200	0.81	49.6	0.1	Lab	[27]
FG	PSC	200	0.79	65.9	0.1	Lab	[27]
(TCO)	a.Si	300	8	_	30	Lab	[25]
Glass	a.Si	200	2.34	57.5	3.18	Manufactured (Market)	[26]
PET	DSSC	200	0.54	90.7	0.25	Lab	[26]
FTO/PDOT	GaAs	200	0.87	75	50	Lab	[28]
PET	OPV	200	0.55	56	-	Lab	[43]
PET	Mono c.Si	220	1.63	35.9	2.5	Manufactured (Market)	this work
ETFE	Mono c.Si	220	1.63	35.9	2.5	Manufactured (Market)	this work
EPOXY	Mono c.Si	220	1.93	42.3	2.5	Manufactured (Market)	this work

## Data Availability

This manuscript has associated data in a data repository. All data included in this manuscript are available upon request by contacting the corresponding author.

## Nomenclature and Abbreviations

A	Ampere
CdTe	Cadmium telluride
CIGS	Copper indium gallium selenide
CFLs	Compact fluorescent lamps
DSC	Dye-sensitized solar cell
ETFE	Ethylene–tetrafluoroethylene copolymer
FF	Fill Factor
I_max_	Maximum current
IoT	Internet of Things
I_SC_	Short-circuit current
KΩ	Kilo-ohm
LEDs	Light-emitting diodes
lx	Lumen per square meter
OCA	Optical contact angle
P	Power
P_max_	Maximum electric power
PET	Polyethylene terephthalate
PV	Photovoltaic
R	Load resistance
TCO	Transparent conductive oxide
V	Voltage
V_max_	Maximum voltage
V_OC_	Open circuit voltage
W	Watt
%	Percentage
